# From *In silico* Protein Epitope Density Prediction to Testing *Escherichia coli* O157:H7 Vaccine Candidates in a Murine Model of Colonization

**DOI:** 10.3389/fcimb.2016.00094

**Published:** 2016-08-30

**Authors:** Daniel Tapia, Brittany N. Ross, Anjana Kalita, Mridul Kalita, Christopher L. Hatcher, Laura A. Muruato, Alfredo G. Torres

**Affiliations:** ^1^Department of Microbiology and Immunology, University of Texas Medical BranchGalveston, TX, USA; ^2^Department of Internal Medicine, University of Texas Medical BranchGalveston, TX, USA; ^3^Institute for Translational Sciences, University of Texas Medical BranchGalveston, TX, USA; ^4^Department of Pathology and Sealy Center for Vaccine Development University of Texas Medical BranchGalveston, TX, USA

**Keywords:** *Escherichia coli* O157:H7, immunoinformatics, bioinformatics, vaccine, type III secretion system

## Abstract

Enterohemorrhagic *Escherichia coli* (EHEC) O157:H7 is a leading cause of foodborne illnesses worldwide and is a common serotype linked to hemorrhagic colitis and an important cause of hemolytic uremic syndrome (HUS). Treatment of EHEC O157:H7 infections is complicated, as antibiotics can exacerbate Shiga toxin (Stx) production and lead to more severe symptoms including HUS. To date, no vaccines have been approved for human use, exposing a void in both treatment and prevention of EHEC O157:H7 infections. Previously, our lab has shown success in identifying novel vaccine candidates via bio- and immunoinformatics approaches, which are capable of reducing bacterial colonization in an *in vivo* model of intestinal colonization. In this study, we further characterized 17 of the identified vaccine candidates at the bioinformatics level and evaluated the protective capacity of the top three candidates when administered as DNA vaccines in our murine model of EHEC O157:H7 colonization. Based on further immunoinformatic predictions, these vaccine candidates were expected to induce neutralizing antibodies in a Th2-skewed immunological response. Immunization of BALB/c mice with two of these candidates resulted in reduced bacterial colonization following EHEC O157:H7 challenge. Additionally, immune sera was shown to prevent bacterial adhesion *in vitro* to Caco-2 cells. Together, this study provides further validation of our immunoinformatic analyses and identifies promising vaccine candidates against EHEC O157:H7.

## Introduction

Enterohemorrhagic *Escherichia coli* (EHEC) O157:H7 is a gram-negative bacteria, member of the Shiga-toxin producing pathogroup of *E. coli* (STEC) strains. Infections with EHEC O157:H7 are associated with diarrheal episodes, including severe manifestations such as hemorrhagic colitis and hemolytic uremic syndrome (HUS) (Nguyen and Sperandio, 2012). Ingestion of *E. coli* O157:H7 bacteria is primarily through the consumption of contaminated food (animal and produce) products and water. The *E. coli* O157:H7 serotype is responsible for ~0.9 cases of hemorrhagic colitis per 100,000 in the United States, and an estimate 79,420 total cases each year (Marks et al., 2013). In addition, *E. coli* O157:H7 infections result in a significant number of hospitalizations each year; complications predominantly affecting children and elderly patients (Marks et al., 2013). According to a 2013 report by the United States Department of Agriculture (USDA), *E. coli* O157:H7 alone cost ~272 million dollars in hospitalizations and corporate food-related losses (Batz et al., 2014). Moreover, enteric infections caused by O157:H7 lead to a 2–5% mortality rate in children with HUS (Banatvala et al., 2001; Rangel et al., 2005). Worldwide, the highest rate of HUS occurs in Argentina where 400 new cases are reported annually, and the frequency of HUS ranges from 10 to 17 cases per 100,000 children (Rivas et al., 2006). This rate is an overwhelming 10-fold difference compared to most developed countries (Rivas et al., 2006).

*E. coli* O157:H7 infections are mediated primarily by the interaction between intimin (Int) adhesin and its cognate translocated intimin receptor (Tir) (Kenny et al., 1997; Deibel et al., 1998). The type-III secretion system (T3SS) functions in delivering Tir and other effector molecules into the host cytoplasm of the gut epithelial cells, resulting in host cytoskeletal rearrangement and the formation of distinct, pedestal-like structures (McDaniel et al., 1995; Elliott et al., 1998; Campellone et al., 2004). These changes are characteristic of a lesion known as attaching and effacing (A/E), which are a hallmark in the intimate attachment of bacteria to the mucosal epithelia (Nataro and Kaper, 1998).

There are currently two EHEC vaccines available for animal use; however, to date, there are no vaccines approved for use in humans (Snedeker et al., 2012; Varela et al., 2013). EHEC infections remain a significant clinical challenge, antibiotic use is contraindicated due to the ability to exacerbate Shiga toxin (Stx) production and increasing the risk of HUS (Karch et al., 1999; Matsushiro et al., 1999). Consequently, it remains an important task to identify vaccine candidates capable of reducing bacterial colonization capabilities that will prevent further complications in humans.

Our lab has previously developed an optimized bioinformatic screening method to identify EHEC O157:H7 vaccine candidates not present in commensal *E. coli* flora (García-Angulo et al., 2013, 2014; Kalita et al., 2014). Using a stepwise screening approach, we down selected from the entire EHEC O157:H7 proteome and identified priority vaccine candidates, which were chosen based on desirable physiochemical properties, antigenicity, and MHC affinity. We then validated our screening methodologies *in vivo* by immunizing mice with pVAX-encoded candidates with high, medium, and low predicted antigenicity. The candidates of high predicted antigenicity produced higher antibody titers, decreased bacterial shedding, and reduced cecal and large intestine murine colonization (Kalita et al., 2014). Taken together, these observations suggest that these vaccine candidates are capable of inducing neutralizing antibodies that can effectively prevent the attachment of *E. coli* O157:H7 to intestinal epithelial cells. It's important to note that some of the high priority (HP) candidates identified are predicted to play a role in the structure or function of the T3SS (García-Angulo et al., 2014). Because the T3SS is critical to EHEC O157:H7 virulence and pathogenesis, the inclusion of these antigens into a subunit vaccine could prove valuable in generating a protective immune response.

Our previous study tested randomly pooled candidates *in vivo* and found that immunization with a truncated T3SS structural *escC*, resulted in the most significant reduction in bacterial load (García-Angulo et al., 2014). The *escC* gene (locus AE005596_9) encodes for a structural protein of the T3SS that forms a polymer ring spaning the bacterial outer membrane (Sekiya et al., 2001; Spreter et al., 2009; Tree et al., 2009). In our present study, we sought to identify comparable candidates by screening the density of immunogenic epitopes within our highly ranked proteins. By performing a mature epitope density (MED) prediction, we were able to cluster candidates based on similarities in putative immunogenic Th2 epitopes (MED_Th2_). This method allowed us to identify two new candidates, *lomW* (locus AE005298_8), a gene ecoding a putative outer-membrane protein belonging to the Lom precursor of a bacteriophage Bp-933W, and *escJ* gene (locus AE005514_9), encoding a putative lipoprotein associated with the T3SS. These candidates were cloned in pVAX1 vector and administered intranasally to BALB/c mice. Upon vaccination, we observed the largest increase in sIgA levels from *lomW* in comparison to *escC* and *escJ*. While no significant difference was detected in total IgG levels, we did distinguish a significant reduction in bacterial adhesion to intestinal epithelial cells *in vitro* and reduced colonization in a murine model of EHEC O157:H7 infection. Our current study aided in validating and characterizing novel *E. coli* O157:H7-specific vaccine candidates, and defined important immune parameters necessary to prevent intestinal disease caused by *E. coli* O157:H7.

## Materials and methods

### Ethics statement

All manipulations of *E. coli strains* were conducted in approved and certified Biosafety Level 2 facilities at the University of Texas Medical Branch (UTMB), and experiments were performed in accordance with standard operating practices. The animal studies were carried out in strict accordance with the recommendations in the Guide for the Care and Use of Laboratory Animals of the National Institutes of Health. The protocol (IACUC #0709042B) was approved by the Institution for Animal Care and Use Committee at UTMB.

### Bacterial strains culture conditions

All bacterial strains used in this study were stored in 50% glycerol at −80°C. Liquid cultures were generated by inoculating Luria-Bertani (LB) broth, with or without 50 μg/mL streptomycin. Liquid cultures were grown overnight at 37°C with agitation. The prototypical *E. coli* O157:H7 strain 86-24 was used for animal challenge studies. *E. coli* O157:H7 wildtype strain EDL933 was utilized for gene amplifications and bacterial adhesion assays. *E. coli* strain DH5α (Life Technologies) was used for propagation of strains bearing pVAX1 plasmid and was routinely grown in LB broth and agar containing kanamycin (Sigma, 50 μg/mL) for plasmid selection.

### Immunoinformatic analysis

We used NetMHC-II (Nielsen and Lund, 2009), NetCTL (Larsen et al., 2007) and ABCPred (Saha and Raghava, 2006) to predict the MHC class II, MHC class I and linear B-cell binding epitopes, respectively, in EHEC O157:H7 specific proteins (further details in García-Angulo et al., 2014; Kalita et al., 2014). Program outputs reported the binding affinity (IC_50_) of individual epitopes to various HLA alleles. Based on these IC_50_ values, all possible 9-mer epitopes from each protein were predicted as weak (IC_50_ > 500 nM) or strong binding (IC_50_ > 50 nM). We selected only strong binding peptides having a predicted IC_50_ value < 50 nM (used as threshold). To assess the potential to induce a Th2-skewed protective response, we focused on NetMHCII output consisting of six HLA-DP, six HLA-DQ, and 14 HLA-DR alleles. We derived two parameters from the NetMHCII output: a) average MHC Affinity (Avg-MA) for all epitopes in a given protein sequence and b) mature epitope density (MED_Th2_) score using the formula below, where epitope length is core 9-mer (Santos et al., 2013).

(1) $$\begin{array}{r} {\text{MED}}_{\text{Th}2}=\frac{\text{Predictions}}{\text{Chances}}\\ =\frac{\text{number of predicted epitopes X }\left(50-\text{Avg-MA}\right)}{\text{protein sequence length}-\text{epitope lengt}{\text{h}}^{*}\;+\;1} \end{array}$$

This MED_Th2_ score reflects aggregate T-cell (Th2) epitope content. Higher scores indicate a better prediction for the protective nature of the protein. Also, the number of alleles bound by each protein was evaluated in order to target a larger population coverage. A heatmap of Avg-MA against each HLA-allele for all protein sequences was generated (Supplemental Figure 1). A clustered image map (CIM) of a normalized matrix was created that correlates Avg-MA of each allele to different proteins. For each protein, mean and standard deviation were calculated from their Avg-MA for all alleles. Z-score transformation was calculated for each of the alleles by subtracting each Avg-MA value by the row mean and dividing by the row standard deviation (Kalita et al., 2013). Hierarchical clustering of HP proteins was performed using an average-linkage clustering algorithm based on their MED_Th2_ scores.

### DNA vaccine construction

Vaccine candidates were amplified from *E. coli* EDL933 genomic DNA. Forward (Fw) and reverse (Rv) primer sequences contained *Hind*III and *Xho*I restriction sites, respectively (Supplemental Table 1). The 5′ end of Fw primers were designed with a Kozak consensus sequence (ACCATGG) to enhance transcription. Genes were amplified with Phusion® High Fidelity Polymerase (New England Biolabs) and ligated into the eukaryotic expression vector pVAX1 (Invitrogen, Life Technologies). Plasmids containing desired candidates were verified by directional sequencing and transformed into competent *E. coli* DH5α for propagation. For immunization studies, plasmids were purified using the Endotoxin-free Giga Kit (Qiagen) according to manufacturer's instructions. DNA samples were quantified using a Epoch Microplate Spectrophotometer (BioTek) and stored at −20°C.

### Immunization and sample collection

Six to eight-week-old female BALB/c mice were obtained from Charles River Laboratories and housed in a specific pathogen-free barrier under biosafety level 2 conditions and allowed to acclimate for 5 days prior to vaccination. Mice were divided into 5 groups (*n* = 10 each), including pVAX (vector), *lomW* (pVAX-10), *escJ* (pVAX-41), *escC* (pVAX-56), and combination (pComb). Mice were anesthetized using isoflurane inhalation and administered a prime and two boosts (days 0, 14, and 28) intranasal (i.n.) immunization of ~60 μg DNA in Tris-EDTA. Prime vaccinations were administered along with Cholera Toxin (CT) as adjuvant (1 μg/uL). In the case of the combination vaccine, ~20 μg of each plasmid were mixed and administered as a single vaccine. Fecal and sera samples were collected prior to vaccination for determination of baseline antibody titers. Fecal samples were collected following final boost to monitor mucosal antibody titers. Briefly, fecal pellets were weighed and diluted to 1 g/mL in PBS. After homogenization by vortexing, fecal samples were then centrifuged at 4000 rpm for 10 min. Supernatants were stored at −20°C prior to IgA measurement. Sera samples were collected 2 weeks after prime and second boost vaccination to monitor changes in antibody levels. Sera was collected via retro-orbital bleeding and incubated at room temperature for 30 min to allow clotting. Sera was separated from whole blood by centrifugation at 10,000 rpm for 10 min. Supernatants were collected and stored at −80°C prior to enzyme-linked immunosorbent assay (ELISA).

### Infection

Two weeks after the second boost, all mice were challenged with a dose of 5 × 10^9^ CFU of streptomycin resistant *E. coli* O157:H7 strain 86-24 via gavage (400 μL). Food was restricted 12 h before infection but was administered *ad libitum* throughout the remainder of the study. Two hours prior to challenge, mice were injected intraperitoneally with cimetidine (50 mg/kg, Sigma) to reduce stomach acidity. Fecal samples were collected daily for 7 days to assess bacterial shedding. Fecal pellets were homogenized in phosphate-buffered saline (PBS), serially diluted, and plated on MacConkey agar plates containing streptomycin (25 μg/mL) and incubated at 37°C. To enumerate bacterial colonization in gastrointestinal tract, mice were euthanized, and ceca and large intestines were removed. Organs were homogenized in 1 mL PBS, serially diluted and plated on MacConkey agar containing streptomycin.

### ELISA

Total IgG and IgA responses were determined using Ready-set-Go! ELISA kits (EBioscience) and were performed according to manufacturer's instructions. To determine immunoglobulin levels, polystyrene 96-well high-binding ELISA plates (Nunc, Denmark) were coated overnight with capture IgG or IgA antibody at 4°C. The plates were washed 2x with PBS containing 0.05% Tween 20 (PBS-T) prior to blocking in 2X Assay Buffer. For IgG, the serum samples were diluted (1:1000 and 1:10,000) in 1X Assay Buffer. Similarly, for IgA levels, samples were diluted (1:2 and 1:4) in 1X Assay Buffer. Following incubation, horseradish peroxidase (HRP)-conjugated goat anti-mouse IgG, or goat anti-mouse IgA was diluted in 1X Assay Buffer (1:250) and added to ELISA plates to determine IgG and IgA concentration, respectively. Plates were incubated with agitation for 3 h at room temperature, followed by washing. A total of 100 μL of tetramethylbenzidine (TMB) was added to each well and incubated at room temperature for 15 min. The reaction was stopped using 100 uL of 2N H_2_SO_4_ and plate was read at 450 nm (Biotech Microplate Spectrophotometer).

### Bacterial adhesion assay

Caco-2 cells (ATCC® HTB-37™) were maintained at 37°C with 5% CO_2_ in complete HTB-37 medium. Complete HTB-37 media consisted of Eagle's Minimum Essential Medium (EMEM, GIBCO) supplemented with 2 mM glutamine, 1 mM sodium pyruvate, 1X non-essential amino acids, penicillin-streptomycin (100 U/ml, 100 μg/ml), and 10% fetal bovine serum. For adhesion assays, 12-well plates were seeded with 10^5^ cells per well and incubated as described above to achieve 80% confluence. Approximately 1 h prior to infection, the monolayer was washed twice with 1 ml PBS prior to addition of 1 ml medium containing no supplements. Fresh bacterial culture of *E. coli* O157:H7 strain EDL933 was grown in LB overnight at 37°C prior to infection. Bacterial culture was diluted in LB (1:100) and incubated at 37°C, shacking, until culture reached an OD_600_ of 1.0. Culture was pelleted at 5000 × g for 5 min, resuspended in PBS (Ca^2+^ and Mg^2+^ free), and plated for input bacterial load. Remaining bacteria was incubated with immune or naïve sera (5 and 10%) for 45 min at 37°C with agitation. At this time, media was removed and replaced with 1 ml fresh media containing 10^7^ bacterial cells (multiplicity of infection [MOI], 100). Inoculated monolayers were incubated for 3 h at 37°C with 5% CO_2_. After incubation, cells were washed three times with PBS prior to addition of 200 μl of 0.1% Triton X-100 in PBS. Wells were incubated at 37°C until cell monolayer detached from the plate. Monolayers were homogenized by pipetting, then samples were serially diluted and plated onto LB agar. The percentage of bacteria recovered was calculated as the number of CFU/ml recovered divided by the input CFU/ml to account for slight variances in input between groups.

### Statistical analysis

Statistical significance between control and vaccinated groups was assessed using GraphPad software. One-way analysis of variance (ANOVA) and Student *t* test were used to analyze the data for colonization and antibody response, respectively. Adhesion assay experiments were repeated in triplicate. Bacteria recovered were normalized to the mean percentage of the bacteria inoculated and the groups were compared using one-way ANOVA followed by Kruskal-Wallis *posthoc* test. *P* < 0.05 were considered significant.

## Results

### Clustering of vaccine candidates by immunoinformatic analysis

**Figure 1:**
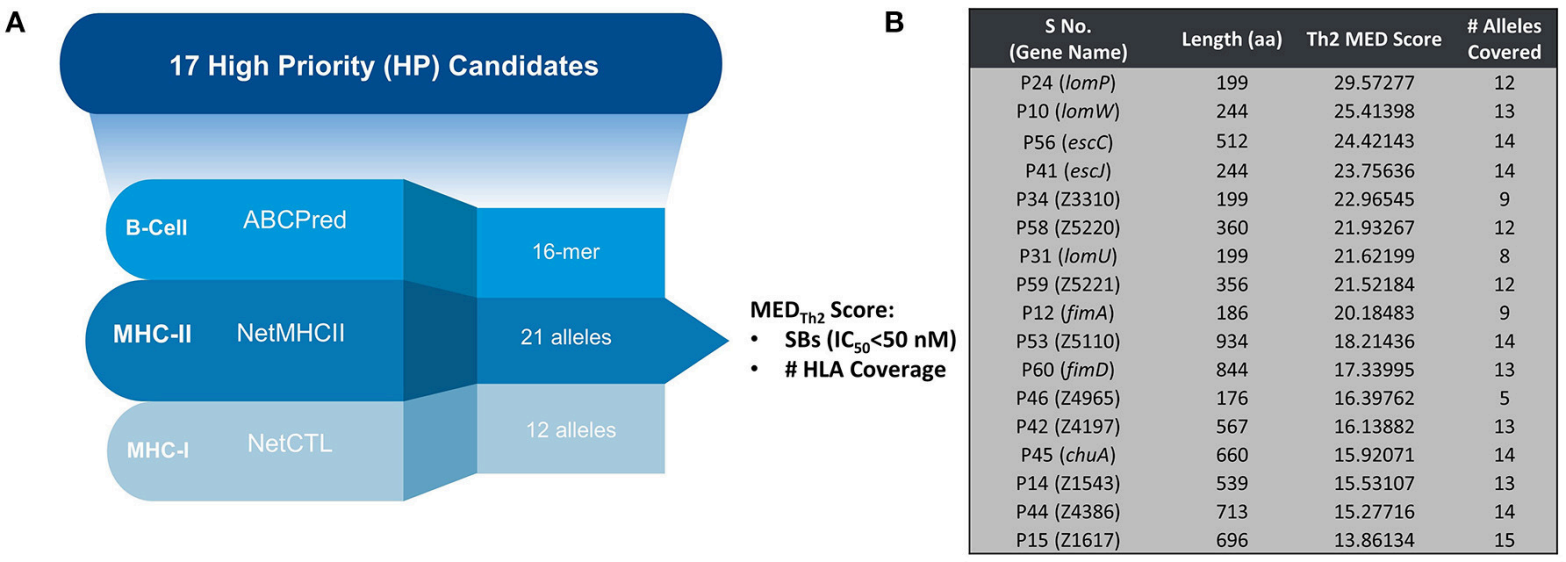
**Classification of identified candidates**. **(A)** Schematic representing prediction strategy for candidate selection of HP candidates. **(B)** Table showing individual candidates along with their compiled MED_Th2_ score and allele coverage across 17 high-priority candidates. The input to the equation was MHC-II prediction results (NetMHCII) only. Hence, it is expected to provide us with Th2-oriented inference.

**Figure 2:**
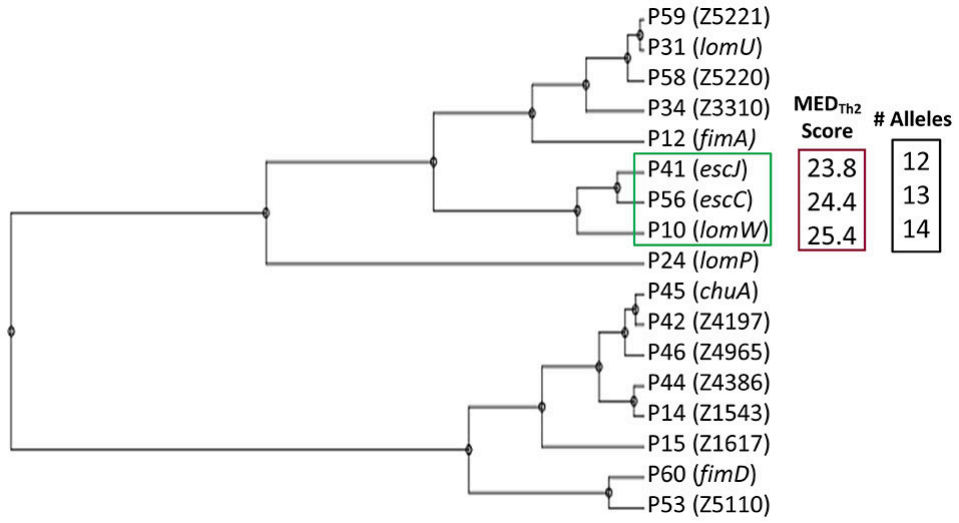
**Dendrogram of vaccine candidates**. Clustering of candidates based on Mature Epitope Density (MED) Score. Selected candidates (green), their compiled MED_Th2_ score (red) and the number of HLA alleles covered based (black) are highlighted.

Figure 1A provides a schematic representation of Th2-oriented epitope prediction and selection of seventeen, potentially protective, high-priority (HP) vaccine candidates in EHEC O157:H7 (García-Angulo et al., 2014). The distribution of high-binding epitopes across numerous HLA alleles is depicted in a heatmap (Supplemental Figure 1). This heatmap highlights allele coverage, with proteins exhibiting increased allele coverage, and thus more likely to induce an effective immune response in a heterogenous population. While the majority of high priority (HP) proteins have high MED_Th2_ scores (above 10, Figure 1B), there is selective binding across the array of HLA alleles. For example, while *lomW* showed a high predictive binding affinity toward DQ5 and DRB6 alleles, the *escJ* epitopes may bind more strongly to DP4 as compared to other gene products. The data in panel 1B was used to derive a dendrogram (Figure 2) to further highlight protein clustering based on MED_Th2_ score. Based on our previous study that tested a pool of three randomly selected HP candidates, immunization with truncated *escC* resulted in the most significant reduction in bacterial colonization. However, that study utilized only the second half of the gene as a vaccine target instead of the entire gene, because we had problems to clone it. In our present study, we were successfully able to clone the full-length *escC* gene. The goal of this study was to examine the protective capacity of whole *escC* gene together with two closely related genes (*lomW* and *escJ*). In summary, candidates were selected based on MED_Th2_ scores, allele coverage, physiochemical features, and predicted function.

### Bacterial colonization in immunized mice with DNA vaccine

**Figure 3:**
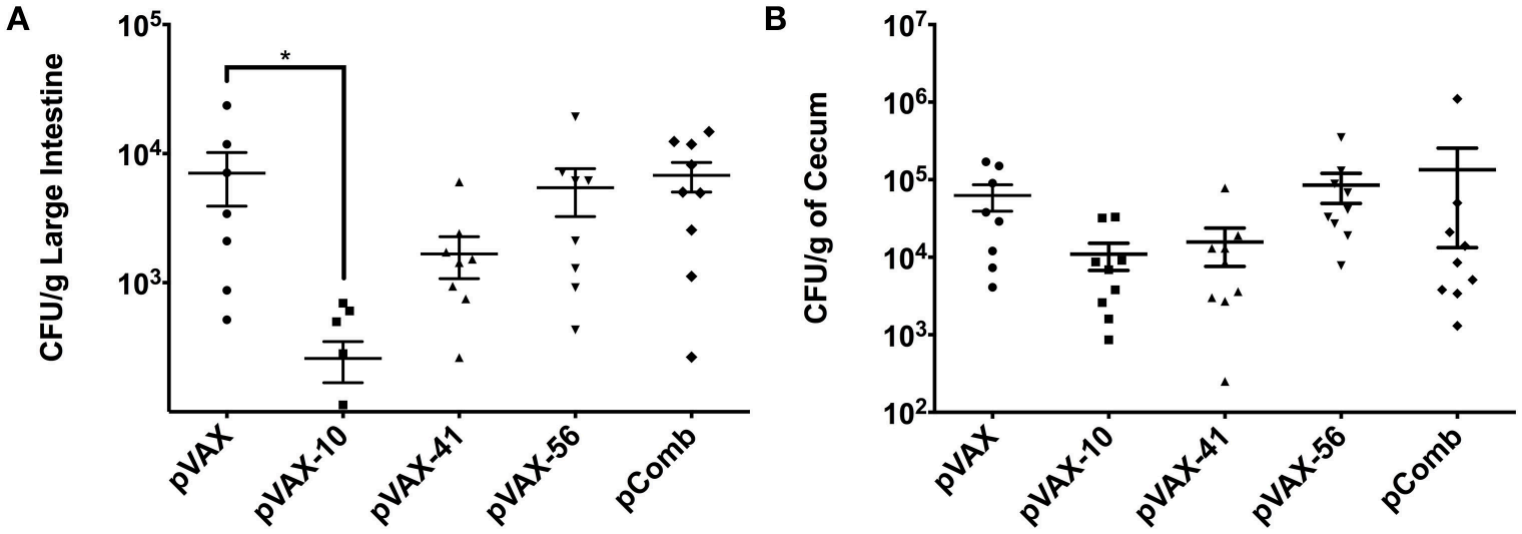
**Bacterial counts in infected mice with EHEC O157:H7**. Bacterial colonization of large intestine **(A)** and cecum **(B)** segments as collected from mice vaccinated with pVAX1, *lomW* (pVAX-10), *escJ* (pVAX-41), *escC* (pVAX-56), and pComb followed by challenge with 5 × 10^9^ CFU of EHEC O157:H7. Bacterial counts are represented as CFU per gram of tissue. Means ± the SEM of the CFU/g from 10 mice presented and an asterisk (^*^) indicates statistical significance as defined (*p* < 0.05).

BALB/c mice were immunized with the DNA vaccine candidates as desbribed in Material and Methods. Two weeks after the last immunization, animals were challenged with a dose of 5 × 10^9^ CFU of streptomycin-resistant *E. coli* O157:H7 strain 86-24 via gavage. Seven days post-challenge, large intestines and ceca were collected to enumerate bacterial colonization. The bacterial load in the gastrointestinal tract (Figure 3) indicates bacterial reduction in mice immunized with the three tested candidate groups *lomW* (pVAX-10), *escJ* (pVAX-41), *escC* (pVAX-56) compared to pVAX-only immunized group. When a combination of all three (pComb) candidates were administered, the reduction in colonization was minimum. Immunization with *lomW* (pVAX-10) resulted in the greatest reduction in bacterial colonization in the large intestine (*p* = 0.0423) (Figure 3A). A similar trend is observed in cecum colonization, despite no statistical significance (Figure 3B).

### Immune response of mice receiving the DNA vaccine

**Figure 4:**
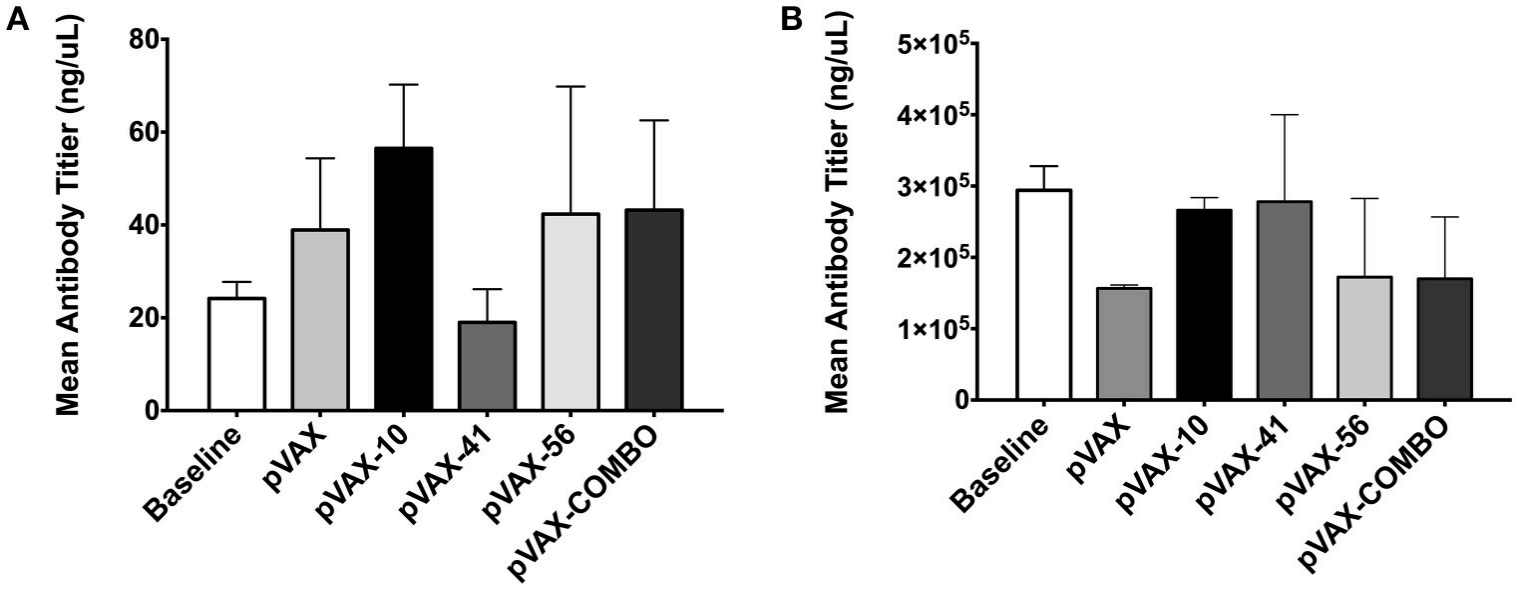
**Immune response from mice immunized with pVAX candidates**. Graphs show secreted immunoglobulin A **(A)** and IgG **(B)** total levels, 2 weeks after last immunization. Mean IgA levels were measured from fecal samples of three immunized mice with *lomW* (pVAX-10), *escJ* (pVAX-41), *escC* (pVAX-56), or pComb. Feces collected prior to immunization (baseline) and of mice immunized with pVAX1 were used as controls. The results are expressed as means ± the SEM of triplicate values obtained from three mice from each group. Statistical significance was defined as (*p* < 0.05). **(B)** Sera collected from mice immunized with vaccine candidates was used to measure total IgG antibodies by ELISA. The results are expressed as means ± of the SEM of triplicate values from three mice in each group.

Fecal samples collected 2 weeks post-immunization were used to measure sIgA production. Mice immunized with *lomW* produced the highest levels of total sIgA when compared to unimmunized mice (baseline), mice immunized with pVAX1 alone, or any of the other immunization groups, though no statistical significance was observed (Figure 4A). Similarly, *escC* was also shown to induce increased sIgA production. Furthermore, sera collected from immunized mice 2 weeks after the last immunization was used to measure total IgG antibodies. Unlike sIgA, there appears to be no observable differences in immunoglobulin production between the immunization groups (Figure 4B).

### Bacterial adherence inhibition by sera

**Figure 5:**
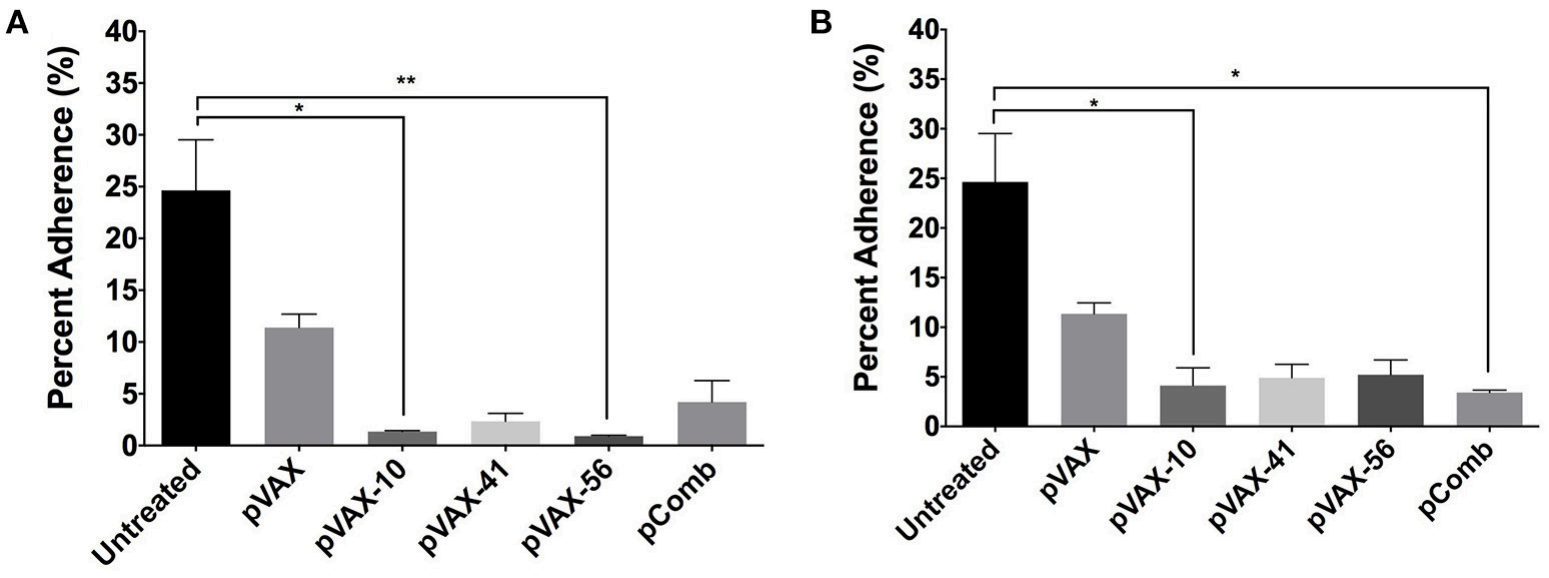
**Bacterial adhesion reduction by immune sera from vaccinated mice**. EHEC O157:H7 serotype EDL933 was incubated with 10% **(A)** or 5% **(B)** pooled sera (*n* = 3) from immunized mice with *lomW* (pVAX-10), *escJ* (pVAX-41), *escC* (pVAX-56), and –pComb in PBS and further incubated with Caco-2 cells at an MOI of 1:100 for 3 h to allow adherence. Sera from pVAX1 immunized mice as well as bacteria alone served as control groups. Bacterial adherence is shown as a percentage of bacteria recovered after incubation. Results are shown as percent adherence and as means ± of the SEM of triplicate values obtained from individual incubation well of bacteria with Caco-2 cells, and an asterisk (^*^) indicates statistical significance as defined (*p* < 0.05), ^**^p < 0.005.

To further characterize the antibodies produced after vaccination, we analyzed the capacity of immune sera to prevent the adherence *in vitro* of *E. coli* O157:H7 to human intestinal epithelial cells (Caco-2). Wild-type *E. coli* O157:H7 strain EDL933 was incubated with pooled sera (*n* = 3) from immunized mice (5 and 10% concentration) at an MOI of 100 prior to infection of Caco-2 cells. We observed the most significant reduction in bacterial adherence from *lomW* (*p* = 0.0466) and *escC* (*p* = 0.0029) immunized mice sera at 10%, and from *lomW* (*p* = 0.0466) and pComb (*p* = 0.0143) at 5% concentration (Figures 5A,B). We noticed a decrease in the percent bacterial adherence from all groups at 5% compared to 10% sera. Also, we noticed a reduction in bacterial adherence by sera from *escJ* and pComb compared to control groups at both concentrations, but this reduction did not reach statistical significance (Figures 5A,B). The ability of sera to inhibit bacterial adherence *in vitro* to intestinal epithelial cells suggest the possibility of some specificity of the sera while recognizing surface-exposed proteins present on EHEC O157:H7 wild-type strain EDL933.

## Discussion

Enterohemorrhagic *E. coli* (EHEC) O157:H7 is a foodborne pathogen and one of the leading causative agents of hemorrhagic colitis worldwide. Because Stx production can be intensified by the administration of antibiotics and is associated with more severe complications, there is a significant need for a human vaccine that can have a positive impact in endemic regions and during outbreaks. To follow up with our vaccine selection, we conducted an immunoinformatic analysis on high-priority (HP) candidates and clustered them to select highly immunogenic candidates with MHC-I, MHC-II, and B-cell epitopes. From our previous experiments that utilized a C-terminal tructated *escC* gene, we noticed a significant decrease in bacterial colonization in both the large intestine and cecum of infected mice. However, in our present study, we noticed that bacterial colonization of mice immunized with the full-length *escC* gene resulted in a less significant reduction compared to the other two candidates tested. This observation may be attributed to the fact that the second half of the gene contains a greater number of epitopes and/or a larger immunodominant segment, that might not be accessible when the full length protein is expressed. However, our present results suggest that immunization with HP candidates identified by the bioinformatic analysis and clustered based on a Th2-skewed immunological response can lead to a bacterial reduction in the large intestine of infected mice. We have identified a putative outer membrane protein Lom precursor from bacteriophage BP-933W (*lomW*), when expressed from a eukaryotic expression plasmid, was able to significant reduce murine intestinal colonization (*p* = 0.0423). Immunization with *lomW* (pVAX-10) was also shown to induce the highest increase in sIgA antibodies compared to baseline levels. The correlation between high sIgA and decreased colonization suggests that sIgA plays an important role in preventing EHEC O157:H7 colonization in the intestinal tract. Moreover, *lomW* has a 13 HLA allele coverage, theoretically allowing for more broad protection. While *escC* (pVAX-56) was not as protective as *lomW*, when tested *in vivo*, sera from immunized mice from both groups was capable of reducing bacterial adherence *in vitro* to primary epithelial cells at 10% sera concentration. Specifically, sera from *lomW* (*p* = 0.0466) and *escC*-immunized mice (*p* = 0.0029) was able to provide the most significant inhibition of bacterial adherence to human epithelial cells. These finding suggests that EscC protein antigens may not be readily accessible when the bacteria is in the intestinal environment, but might still be effective during *in vitro* culture conditions. While we were not able to observe any significant differences in total IgG levels, the role of individual IgG isotypes in mediating protection has not yet been characterized and warrants further attention. The moderate IgG and sIgA antibody titer in response to immunization with pVAX1 alone could, in part, be attributed to variations in individual mouse antibody levels. While reporting individual antibody levels might be a more accurate representation of the results, these levels can vary considerably. Moreover, when previously tested as a truncated gene portion, *escC* was protective. However, in the current study, immunization with the full-length gene provided no significant reduction in bacterial colonization *in vivo*, but was capable of reducing bacterial adherence *in vitro*. These results suggest the possibility that the second portion of the truncated gene may be a good target for vaccine design because its concentration of immunogenic epitopes that might be essential for inducing protective antibody production. As previously stated, it is plausible to propose that expression of the full-length gene might mask immunogenic epitopes or reduce efficacy in antigen presentation.

Currently, one of the most significant obstacles limiting the advancement of a vaccine against EHEC O157:H7 is the potential to disrupt intestinal commensal flora in humans. However, in our selection of candidates and current vaccination study, we took careful steps to avoid selecting genes common to commensal flora. Specifically, we performed genome-wide *E. coli* comparisons to identify genes specific to the most prevalent EHEC O157:H7 serotypes and not present in commensal bacteria. This study combines an *in silico* approach, together with *in vivo* vaccination studies, to evaluate the effectiveness of potential candidates. While the use of a DNA vector as a vaccine is an efficient method to screen a pool of candidates, future studies will focus on evaluating proteins in combination with various platforms and adjuvants to achieve increased protection. Taken together, this study expands the current pool of potential vaccine candidates and provides an important foundation for continued optimization studies toward the development of an effective EHEC O157:H7 vaccine.

## Author contributions

DT, AK, and AT designed research; DT, BR, AK, LM, CH, and MK performed research; DT, AK, LM, and CH analyzed data; and DT, BR, AK, LM, and AT wrote the manuscript.

### Conflict of interest statement

The authors declare that the research was conducted in the absence of any commercial or financial relationships that could be construed as a potential conflict of interest.
