# Supplementary material for: From In silico Protein Epitope Density Prediction to Testing Escherichia coli O157:H7 Vaccine Candidates in a Murine Model of Colonization
Source: Front Cell Infect Microbiol. 2016 Aug 30;6:94. doi: 10.3389/fcimb.2016.00094 (PMC5003871; doi:10.3389/fcimb.2016.00094)
Supplement: Supplementary file 1 [file Table1.DOC]

**Supplemental Table 1**. Primers utilized for construction of vaccine plasmids

| Construct | EHEC Gene insert | Primer | Sequence (5’-3’) |
| --- | --- | --- | --- |
| pVAX-10 | *lomW* (Putative outer membrane protein Lom precursor of bacteriophage Bp-933W) | pVAX10-Fw | 5’-ACC AAG CTT ACC ATG GAG AGT ATA GCA ACA CTG GTT GTG T-3’ |
|  |  | pVAX10-Rv | 5’-ACC CTC GAG TCA GAA TTT CAG GCC AAT GCC AGC-3’ |
| pVAX-41 | *escJ* (Putative lipoprotein of T3SS) | pVAX41-Fw | 5’-ACC AAG CTT ACC ATG GGT GCT GTT GTG CGG CTG CAA G-3’ |
|  |  | pVAX41-Rv | 5’-ACC CCT CGC GTC ACT ACT TAA TTT TCA ACC TGA CT-3’ |
| pVAX-56 | *esc*C (T3SS structural protein) | pVAX56-Fw | 5’-ACC AAG CTT ACC ATG GCA CTA TTT TGC TGC AGT GCA C-3’ |
|  |  | pVAX56-Rv | 5’-ACC AAG CTT ACC ATG GTG CAG TGC ACA AGC TGC C-3’ |
