# Supplementary figures and images for: From In silico Protein Epitope Density Prediction to Testing Escherichia coli O157:H7 Vaccine Candidates in a Murine Model of Colonization
Source: Front Cell Infect Microbiol. 2016 Aug 30;6:94. doi: 10.3389/fcimb.2016.00094 (PMC5003871; doi:10.3389/fcimb.2016.00094)

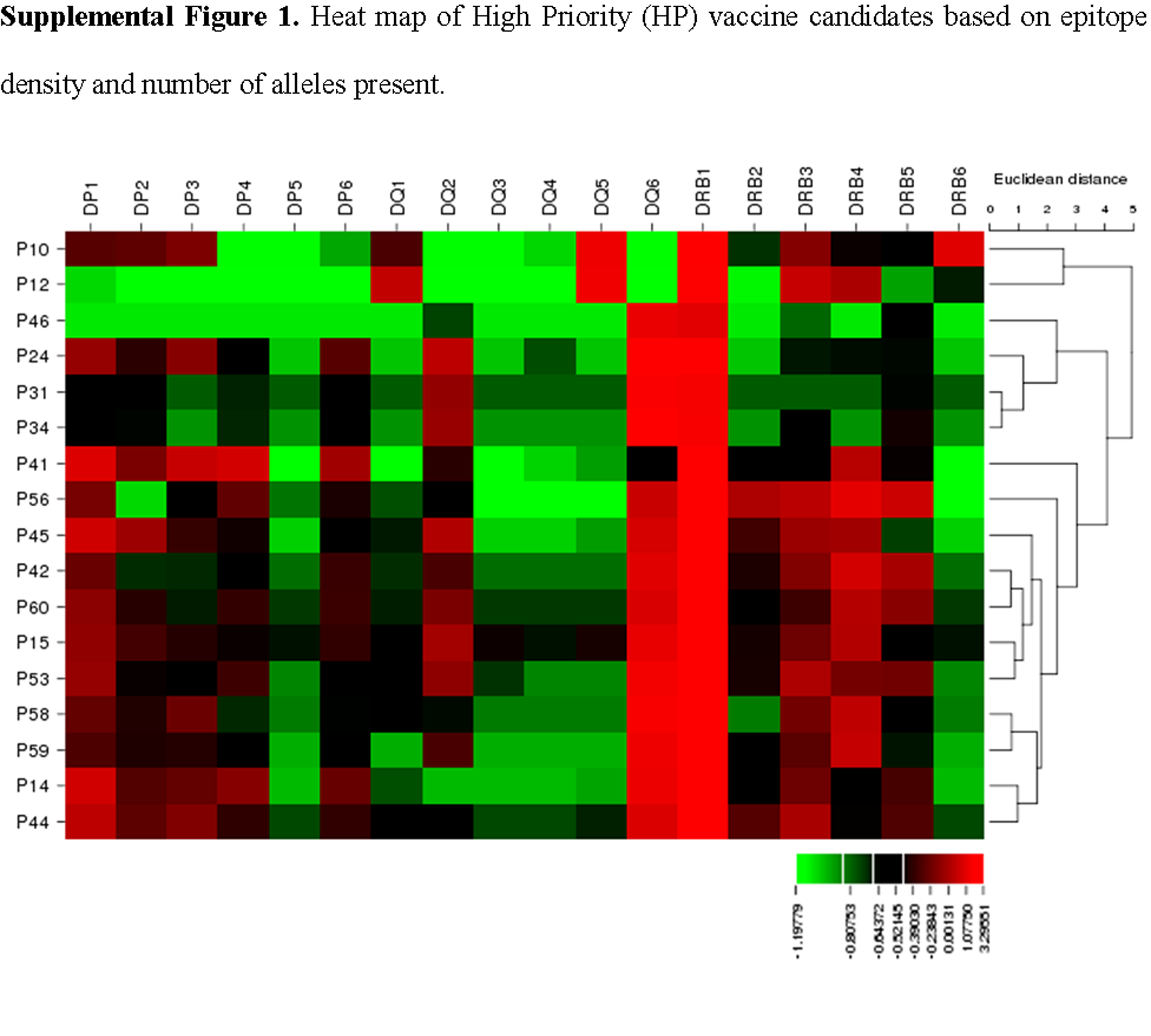

Supplement: Supplementary file 2 [file Image1.TIF]
